# Supplementary material for: Nanostructured Layer of Silver for Detection of Small Biomolecules in Surface-Assisted Laser Desorption Ionization Mass Spectrometry
Source: Materials (Basel). 2022 Jun 8;15(12):4076. doi: 10.3390/ma15124076 (PMC9227941; doi:10.3390/ma15124076)
Supplement: Supplementary file 1 [file materials-15-04076-s001.zip › materials-1720120-supplementary.pdf]

Supplementary materials for the paper entitled

'Nanostructured Layer of Silver for Detection of Small Biomolecules in Surface-Assisted Laser Desorption Ionization Mass Spectrometry'

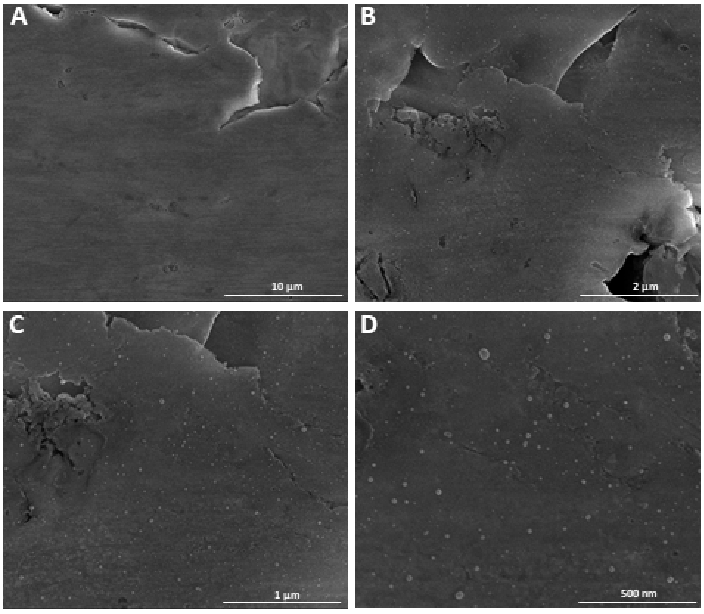

Figure S1. SEM figures of positive electrode (stainless-steel plate) at different magnification values: A –10,000x; B – 50,000x; C – 100,000x; D – 200,000x

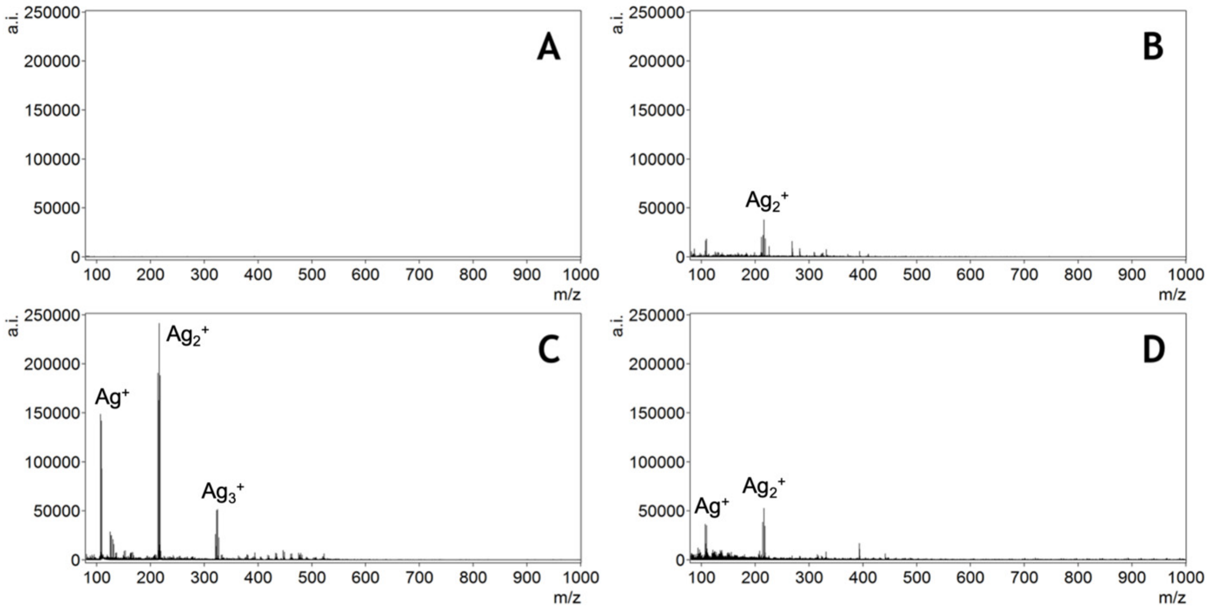

Figure S2. Mass spectra of A – pure steel plate; B – steel plate immersed into AgTFA solution for 15 min; C – negative electrode after electrodeposition from AgTFA (10 V, 15 min) ; D – positive electrode after electrodeposition from AgTFA (10 V, 15 min)

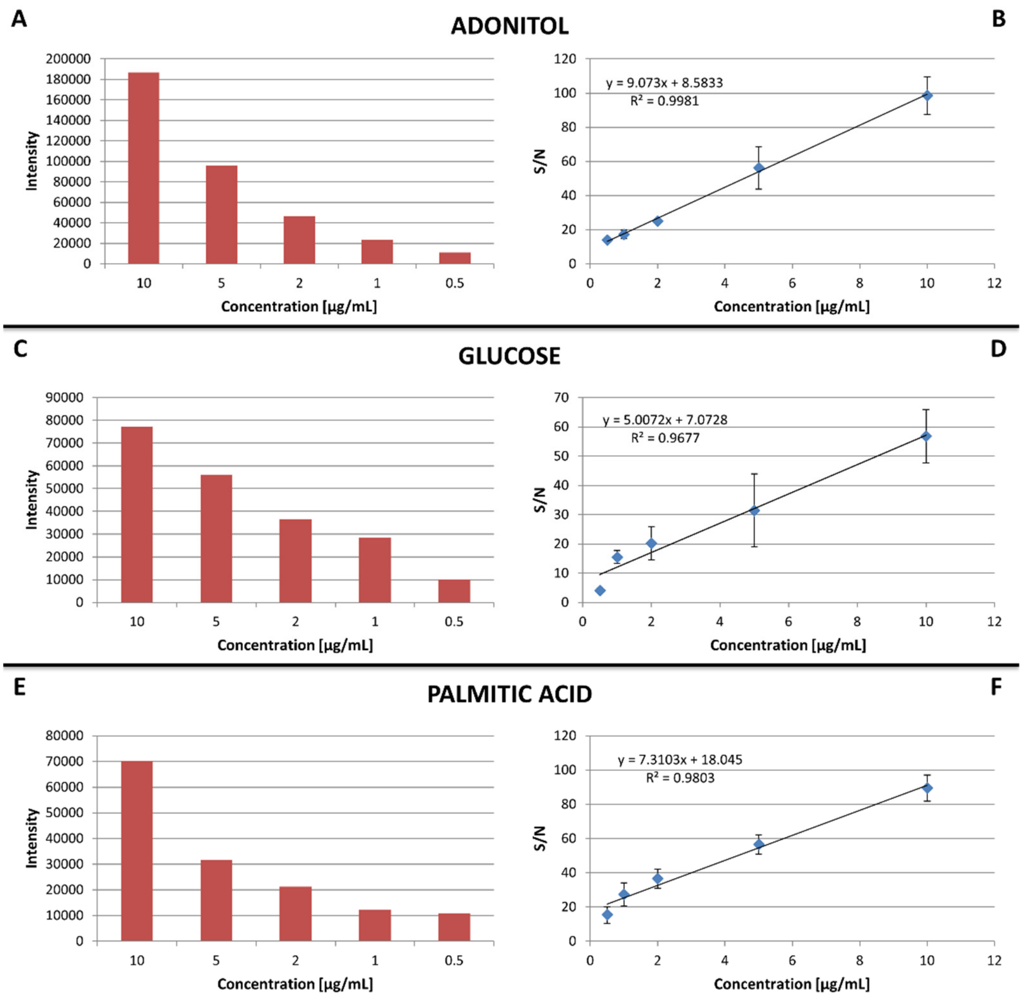

Figure S3. Intensity of $[M+^{107}Ag]^+$ adduct in different concentration (A, C, E) and linear regression equations based on the signal-to-noise (S/N) ratio (B, D, F) for adonitol, glucose and palmitic acid using silver nanostructured layer
